# Corosolic Acid Induces Endothelium-Dependent Vasorelaxation in Isolated Rat Aortic Rings

**DOI:** 10.3390/molecules31162822

**Published:** 2026-08-13

**Authors:** Fangying Chen, Wan Yin Tew, Ming Thong Ong, Mun Fei Yam

**Affiliations:** 1 Institute for Research in Molecular Medicine (INFORMM), Universiti Sains Malaysia (USM), Gelugor 11800, Pulau Pinang, Malaysia; chenfangyingbicf@student.usm.my; 2 Department of Pharmacology, School of Pharmaceutical Sciences, Universiti Sains Malaysia (USM), Gelugor 11800, Pulau Pinang, Malaysia

**Keywords:** Corosolic acid, isolated aortic ring, endothelium, vasorelaxation, potassium channels, calcium signalling

## Abstract

The effect of Corosolic acid (CA) on vascular tone and the possible mecha-nisms involved remain unclear. The SPF-grade male Sprague-Dawley rats (Animal Ethics Approval ID: USM/IACUC/2025/(155)(1406)) were used. The vasorelaxant effects of CA were evaluated in endothelium-intact and endothelium-denuded rings precontracted with phenylephrine (PE), and in endothelium-intact rings precontracted with KCl. Possible mechanisms were examined using L-NAME, ODQ, methylene blue, indomethacin, atropine, propranolol, and potassium channel blockers (glibenclamide, TEA, BaCl_2_, and 4-AP). The effects of CA on voltage-operated calcium channels (VOCCs) and IP_3_ receptor (IP_3_R)-mediated sarcoplasmic reticulum calcium release were also assessed. CA concentration-dependently relaxed endothelium-intact aortic rings precontracted with PE (RMAX = 90.08 ± 7.33%; pD_2_ = 4.21 ± 0.08). The relaxation response was markedly reduced after endothelial removal and in KCl-precontracted rings. EDRF pathway inhibitors (L-NAME, ODQ, methylene blue, and indomethacin) and GPCR-related antagonists (atropine and propranolol) attenuated CA-induced relaxation. Among potassium channel blockers, glibenclamide and TEA showed stronger inhibitory effects. CA did not significantly inhibit VOCC-mediated CaCl_2_-induced contraction but partially reduced PE-induced contraction under calcium-free conditions. The mechanisms for CA treating hypertension may involve NO/cGMP signalling, COX-related prostanoid pathways, GPCR-related mechanisms, potassium channel modulation, and partial inhibition of IP_3_R-mediated calcium release.

## 1. Introduction

Precise regulation of vascular smooth muscle tone plays a central role in maintaining cardiovascular homeostasis, and its dysregulation is closely associated with hypertension, atherosclerosis, and other cardiovascular diseases [1,2,3,4]. Vasomotor responses are mainly regulated by interactions between vascular endothelial cells and vascular smooth muscle cells. Endothelium-derived relaxing factors (EDRFs), such as nitric oxide (NO), prostacyclin (PGI_2_), and endothelium-dependent hyperpolarizing factors, play key roles in vascular relaxation. These signaling molecules regulate vascular smooth muscle contraction and relaxation by activating soluble guanylyl cyclase (sGC), increasing cyclic guanosine monophosphate (cGMP) levels, and modulating potassium channels and calcium signaling pathways [5,6]. In addition, signaling mediated by G protein-coupled receptors (GPCRs) and changes in intracellular calcium concentration are also important regulators of vascular tone [7,8]. Therefore, exploring active compounds that can simultaneously modulate multiple vasomotor regulatory pathways is important for developing new vasodilator drugs.

Natural products have received considerable attention in cardiovascular pharmacology because of their structural diversity and multitarget regulatory properties [9,10]. Corosolic acid (CA), a naturally occurring pentacyclic triterpenoid, is found in a variety of medicinal plants and has been reported to exert diverse biological activities, including anti-inflammatory, antioxidant, antidiabetic, and antitumor effects [11,12,13,14]. Recent studies suggest that CA may protect against cardiovascular diseases by regulating oxidative stress, inflammatory responses, and multiple cellular signaling pathways [15,16]. However, the direct regulatory effects of CA on vascular tone and the underlying molecular mechanisms remain insufficiently studied.

To date, the direct vasorelaxant effect of CA and the relative contribution of endothelial factors, GPCR-related signalling, potassium channels, and calcium-related mechanisms remain unclear. Therefore, this study used isolated rat thoracic aortic rings to evaluate the vasorelaxant effect of CA and to explore the possible pathways involved using pharmacological blockers. The findings are intended to provide preliminary ex vivo evidence on the vascular pharmacological properties of CA.

## 2. Results

### 2.1. Vasorelaxant Effect of CA in Phenylephrine-Precontracted Aortic Rings

**Figure 1:**
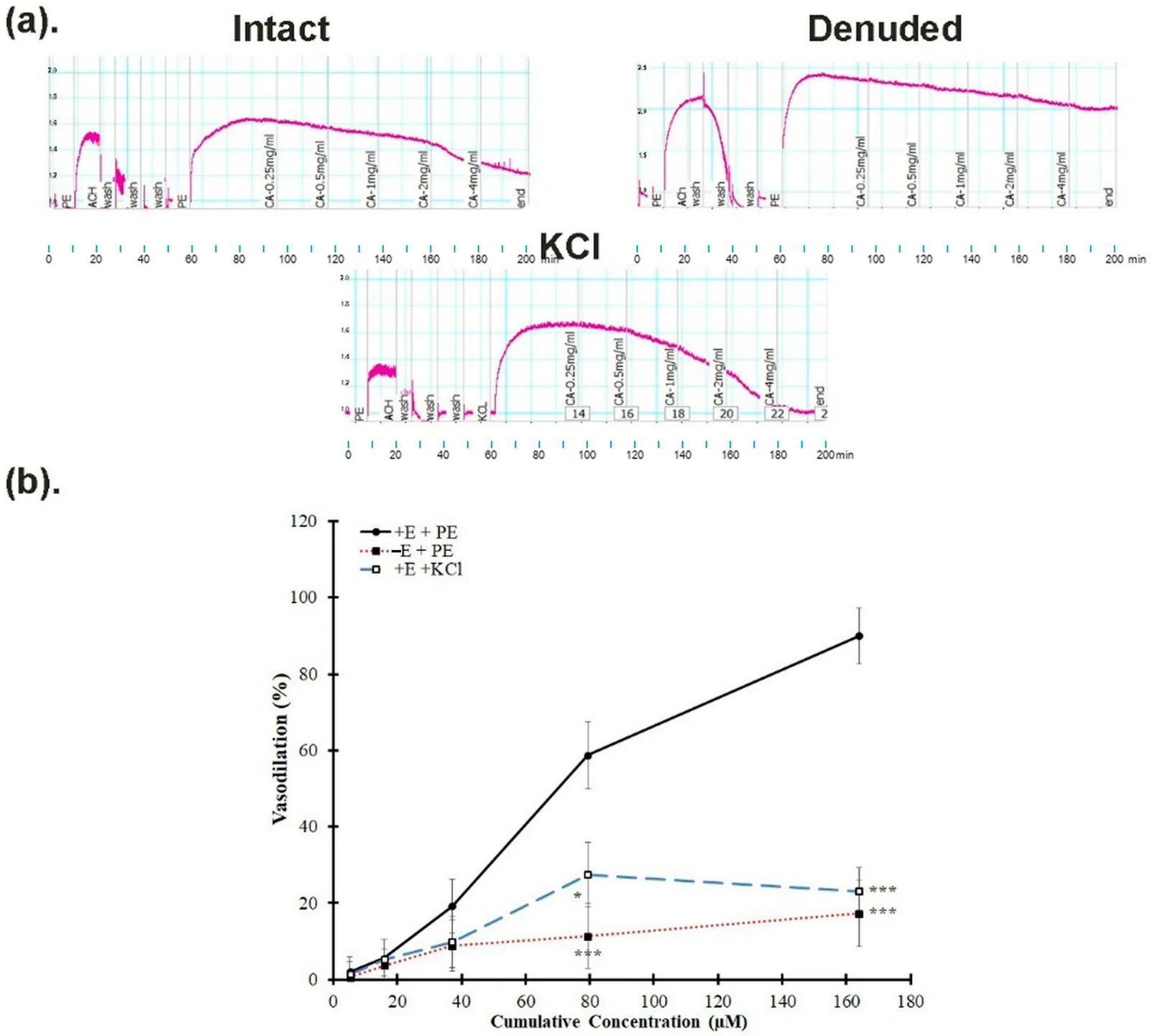
CA induces endothelium-dependent relaxation of PE-precontracted rings. Representative isometric tension traces and concentration-dependent relaxation profiles showing CA-induced vasorelaxation in isolated rat thoracic aortic rings precontracted with phenylephrine in the presence or absence of functional endothelium (+E + PE; −E + PE) or with potassium chloride in endothelium-intact rings (+E + KCl). Data are expressed as mean ± S.E.M. from six independent experiments (*n* = 6), with *n* representing the number of animals/aortic rings. If multiple aortic rings from the same animal were used under the same treatment condition, values were averaged before statistical analysis as described in the Methods. Significance is indicated as * *p* < 0.05, ** *p* < 0.01, and *** *p* < 0.001. (**a**) Representative isometric tension traces under endothelium-intact, endothelium-denuded, and KCl-precontracted conditions; (**b**) concentration-dependent relaxation curves comparing +E + PE, −E + PE, and +E + KCl groups.

As shown in Figure 1 and Table 1, CA exhibited significant vasorelaxant activity at higher cumulative concentrations. The maximum relaxation (RMAX) reached 90.08 ± 7.33%, and the potency (pD_2_) was 4.21 ± 0.08. These results indicate that CA has a marked vasorelaxant effect and support further mechanistic and pharmacological investigations. This condition was used as the control for subsequent related experiments.

### 2.2. Vasorelaxant Effect of CA in Phenylephrine-Precontracted, Endothelium-Denuded Aortic Rings

According to Figure 1 and Table 1, CA induced only limited relaxation in endothelium-denuded aortic rings (RMAX = 17.35 ± 8.61%). Because the relaxation response did not reach 50%, pD_2_ was not determined. This marked reduction compared with endothelium-intact rings indicates that the endothelium is an important contributor to the vasorelaxant effect of CA.

### 2.3. Vasorelaxant Effect of CA in KCl-Precontracted Aortic Rings with Intact Endothelium

As shown in Figure 1 and Table 1, CA produced only weak relaxation in KCl-precontracted aortic rings (RMAX = 23.15 ± 6.17%). Because the relaxation response did not reach 50%, pD_2_ was not determined. The weaker response under KCl precontraction suggests that CA is unlikely to act mainly through direct blockade of voltage-operated calcium entry.

### 2.4. Vasorelaxant Effect of CA on Aortic Rings Following Blockade of EDRF-Related Inhibitors

**Figure 2:**
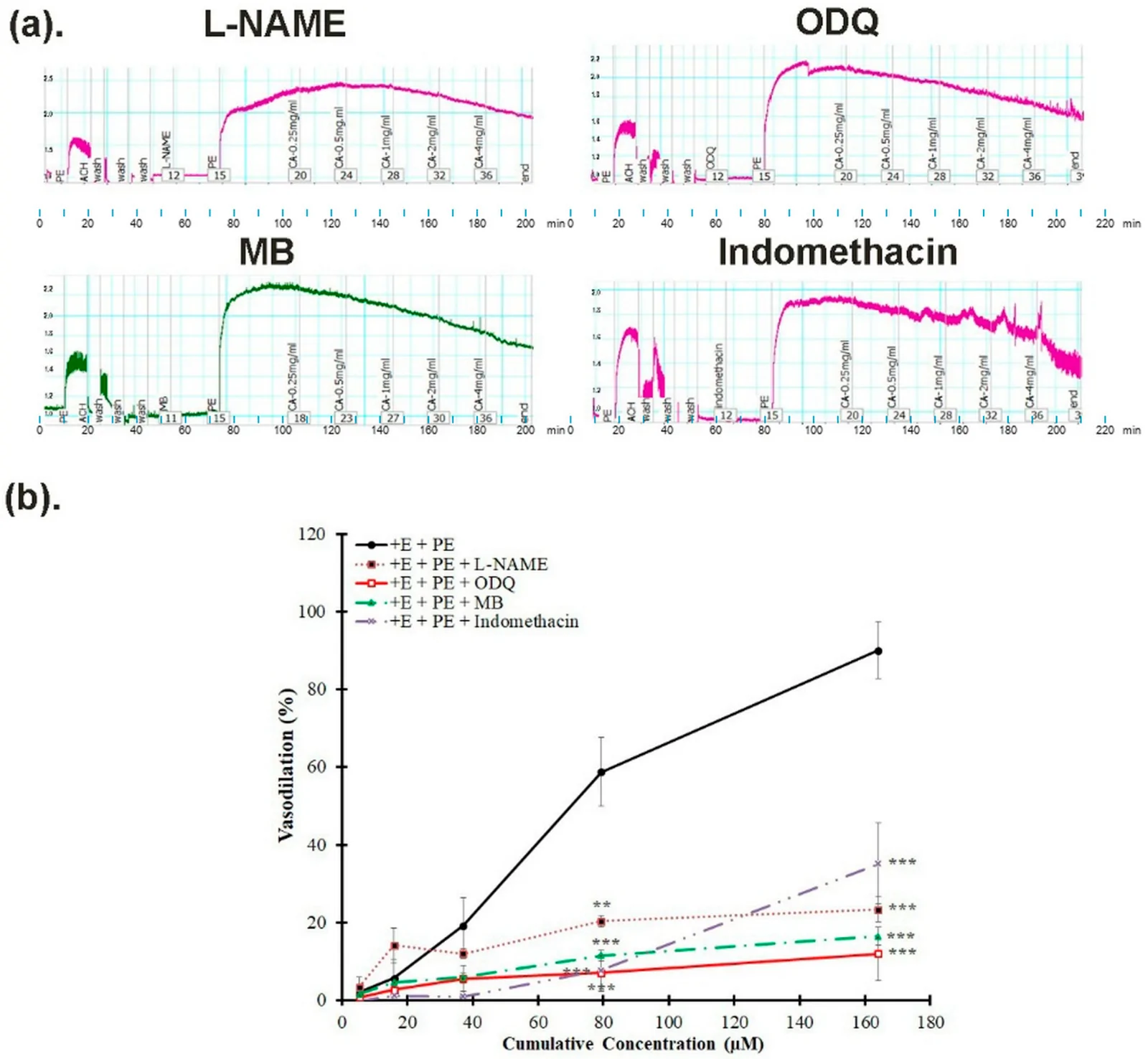
EDRF pathway inhibitors block CA-induced vasorelaxation. Representative isometric tension traces and concentration-dependent relaxation profiles showing CA-induced vasorelaxation in phenylephrine-precontracted isolated rat thoracic aortic rings with intact endothelium (+E) after pretreatment with L-NAME (nitric oxide synthase inhibitor), indomethacin (cyclooxygenase inhibitor), methylene blue (soluble guanylyl cyclase inhibitor), or ODQ (selective soluble guanylyl cyclase inhibitor). Data are expressed as mean ± S.E.M. from six independent experiments (*n* = 6), with *n* representing the number of animals/aortic rings. If multiple aortic rings from the same animal were used under the same treatment condition, values were averaged before statistical analysis as described in the Methods. Significance is indicated as * *p* < 0.05, ** *p* < 0.01, and *** *p* < 0.001. (**a**) Representative isometric tension traces after pretreatment with L-NAME, ODQ, methylene blue, or indomethacin; (**b**) concentration-dependent relaxation curves comparing the EDRF-related inhibitor groups.

As shown in Figure 2 and Table 1, inhibitors of EDRF-related signalling pathways significantly attenuated CA-induced vasorelaxation. After pretreatment with L-NAME, ODQ, methylene blue (MB), and indomethacin, RMAX decreased to 23.38 ± 3.15%, 11.97 ± 6.82%, 16.46 ± 2.33%, and 35.19 ± 10.51%, respectively (all *p* < 0.001). Because these relaxation curves did not reach 50%, pD_2_ was not determined.

### 2.5. Vasorelaxant Effects of CA in Aortic Rings Following Pharmacological Blockade of GPCRs

**Figure 3:**
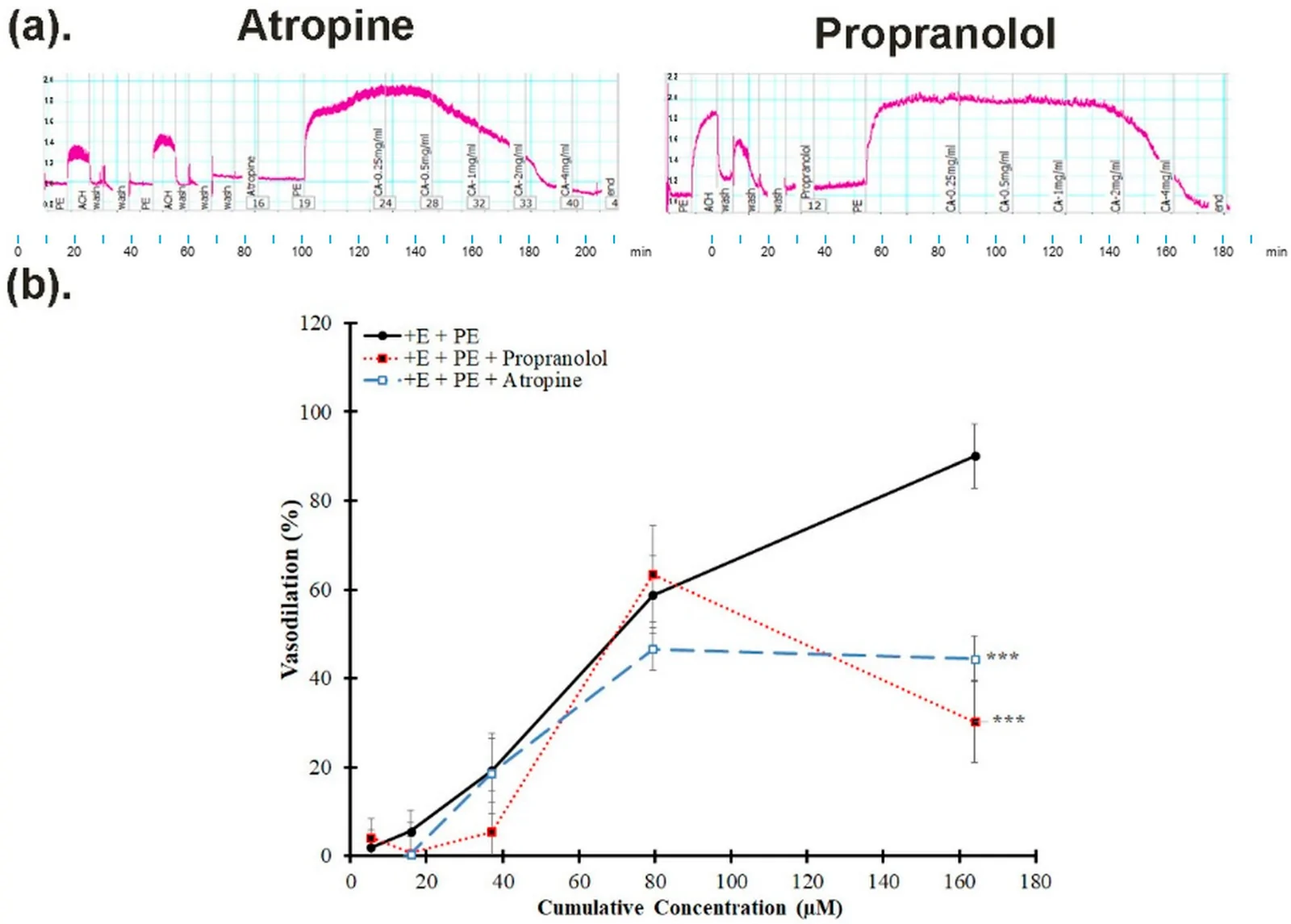
GPCR-related antagonists partially reduced CA-induced vasorelaxation. Representative isometric tension traces and concentration-dependent relaxation profiles showing CA-induced vasorelaxation in phenylephrine-precontracted isolated rat thoracic aortic rings with intact endothelium (+E) after pretreatment with atropine or propranolol. Data are expressed as mean ± S.E.M. from six independent experiments (*n* = 6), with *n* representing the number of animals/aortic rings. If multiple aortic rings from the same animal were used under the same treatment condition, values were averaged before statistical analysis as described in the Methods. Significance is indicated as * *p* < 0.05, ** *p* < 0.01, and *** *p* < 0.001. (**a**) Representative isometric tension traces after atropine or propranolol pretreatment; (**b**) concentration-dependent relaxation curves comparing the GPCR-related antagonist groups.

Figure 3 and Table 1 show that CA-induced vasorelaxation was partially reduced when GPCR-related pathways were inhibited. The inhibition showed a concentration-dependent trend, but the overall effect was less pronounced than that observed after EDRF pathway blockade.

After propranolol and atropine pretreatment, RMAX decreased to 30.23 ± 9.25% and 44.43 ± 5.22%, respectively (both *p* < 0.001). Because both relaxation curves did not reach 50% relaxation, pD_2_ was not determined. These findings suggest that muscarinic- and beta-adrenergic receptor-related signalling may contribute to, but do not prove direct receptor activation by, CA.

### 2.6. Vasorelaxant Effects of CA on Aortic Rings Following Blockade with Different Potassium Channel Inhibitors

**Figure 4:**
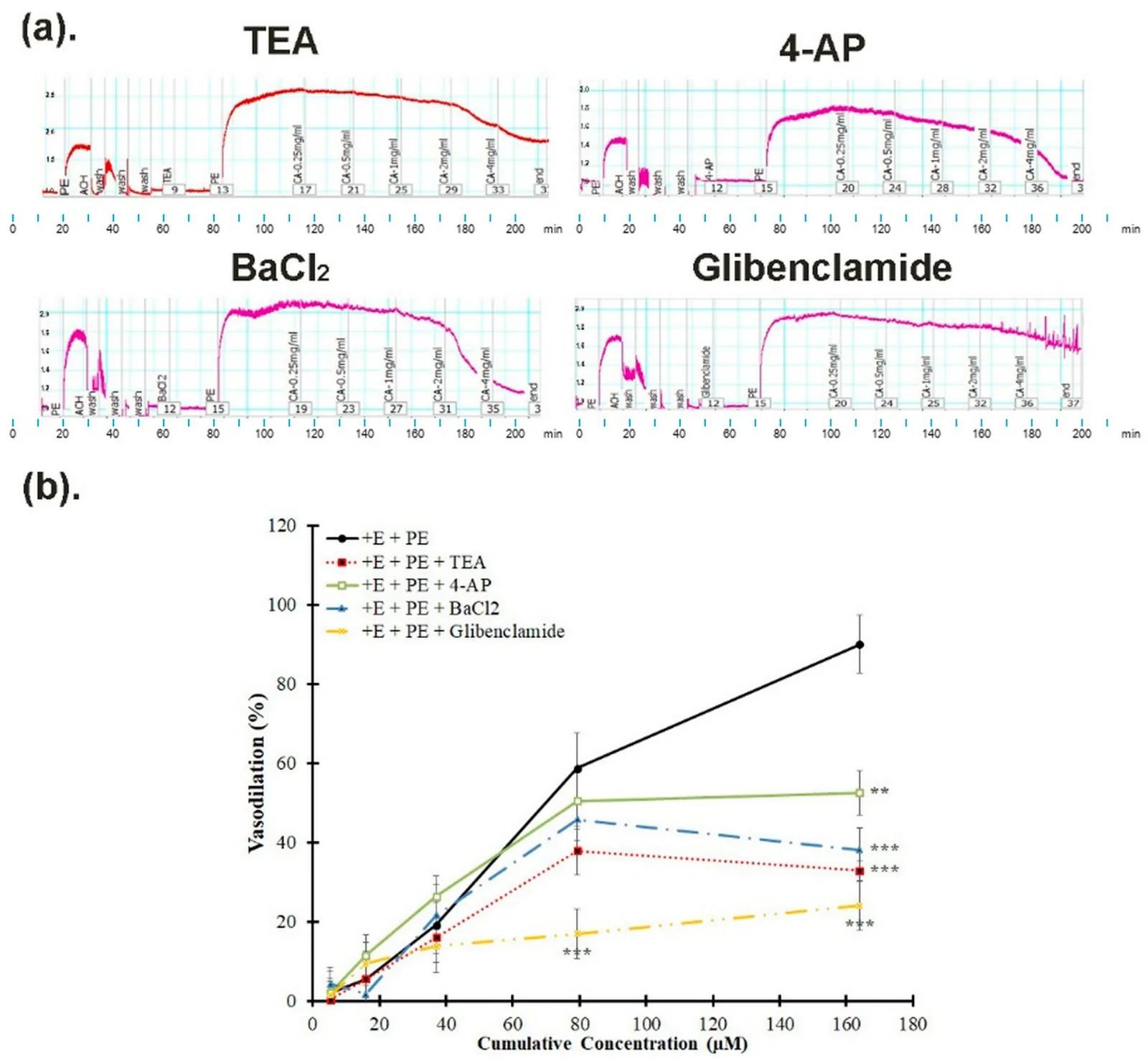
K^+^ channel blockers attenuated CA-induced vasorelaxation. Representative isometric tension traces and concentration-dependent relaxation profiles showing CA-induced vasorelaxation in phenylephrine-precontracted isolated rat thoracic aortic rings with intact endothelium (+E) after pretreatment with TEA, glibenclamide, 4-AP, or BaCl_2_. Data are expressed as mean ± S.E.M. from six independent experiments (*n* = 6), with *n* representing the number of animals/aortic rings. If multiple aortic rings from the same animal were used under the same treatment condition, values were averaged before statistical analysis as described in the Methods. Significance is indicated as * *p* < 0.05, ** *p* < 0.01, and *** *p* < 0.001. (**a**) Representative isometric tension traces after TEA, 4-AP, BaCl2, or glibenclamide pretreatment; (**b**) concentration-dependent relaxation curves comparing the potassium channel blocker groups.

As shown in Figure 4 and Table 1, potassium channel blockers attenuated CA-induced relaxation. Based on blocker sensitivity, glibenclamide and TEA produced stronger inhibition than BaCl2 and 4-AP. These findings suggest that KATP and KCa channels may contribute more prominently to CA-induced relaxation, although direct channel activation cannot be concluded from blocker experiments alone.

### 2.7. Effect of CA on Calcium Channels

#### 2.7.1. Effects on Voltage-Operated Calcium Channels (VOCCs)

**Figure 5:**
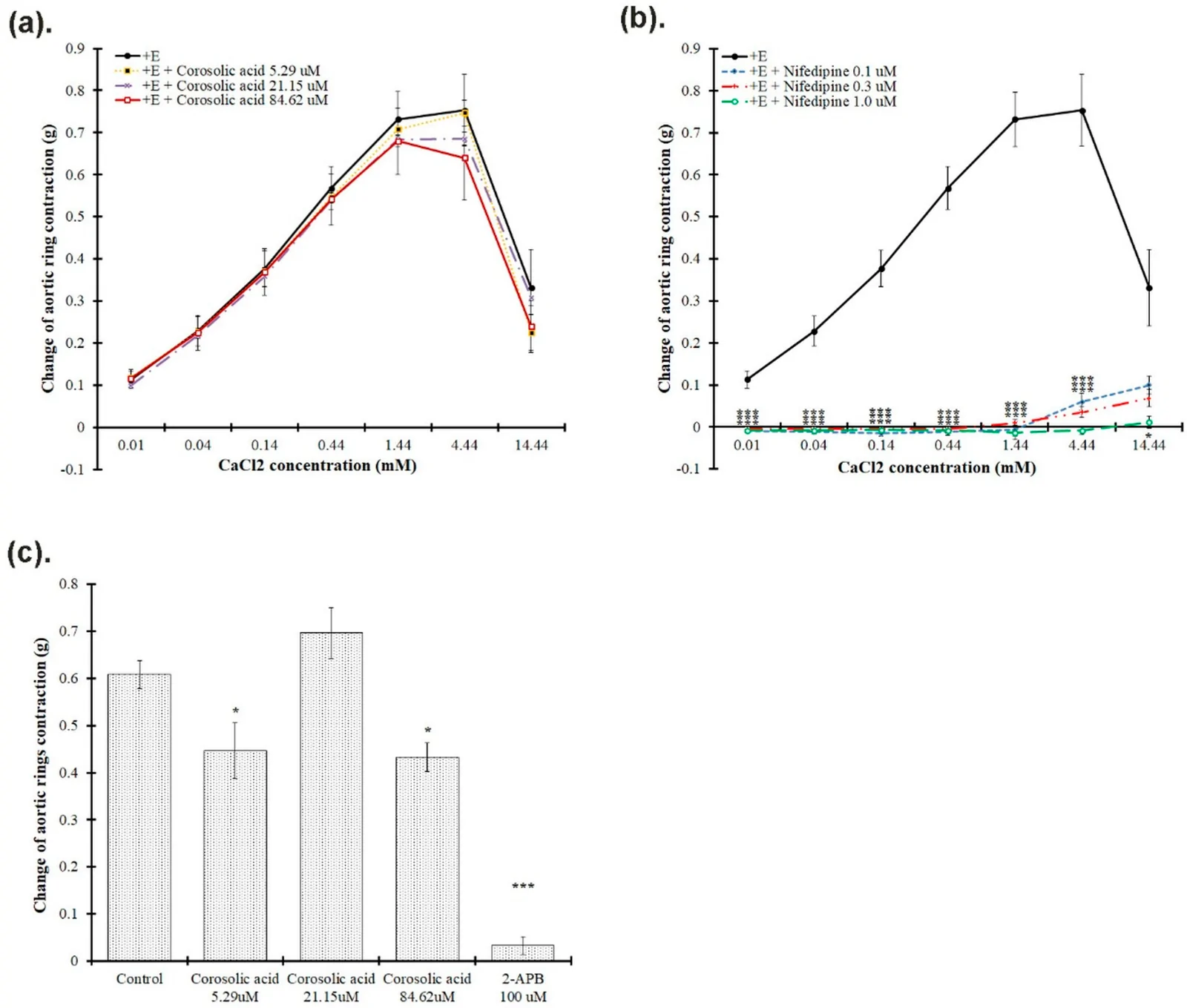
CA inhibits IP_3_R-mediated Ca^2+^ release, not VOCC. Effect of CA (5.29 µM, 21.15 µM, and 84.62 µM) on CaCl_2_-induced vasoconstriction in isolated endothelium-intact aortic rings (**a**,**b**) and on PE-induced contraction in endothelium-denuded aortic rings in Ca^2+^-free KPS solution (**c**). Data are expressed as mean ± S.E.M. from six independent experiments (*n* = 6), with *n* representing the number of animals/aortic rings. If multiple aortic rings from the same animal were used under the same treatment condition, values were averaged before statistical analysis as described in the Methods. Significance is indicated as * *p* < 0.05, ** *p* < 0.01, and *** *p* < 0.001.

As shown in Figure 5a–b and Table 2, cumulative addition of CaCl_2_ induced concentration-dependent vasoconstriction in endothelium-intact aortic rings after VOCC opening in calcium-free, high-K^+^ solution. The positive control nifedipine (0.1 µM, 0.3 µM, and 1 µM) significantly inhibited VOCC-mediated contraction in a concentration-dependent manner (*p* < 0.05), confirming effective VOCC blockade. However, compared with the negative control group, CA (5.29 µM, 21.15 µM, and 84.62 µM) pretreatment did not significantly inhibit CaCl_2_-induced contraction (MAXIMUM CONTRACTION: 0.22 ± 0.14 g, 0.31 ± 0.10 g, and 0.24 ± 0.06 g, respectively; *p* > 0.05).

#### 2.7.2. Effects on Inositol Trisphosphate Receptor (IP_3_R)-Mediated Intracellular Calcium Release

To explore whether CA affects sarcoplasmic reticulum calcium release, experiments were performed under calcium-free conditions. As shown in Figure 5c and Table 2, phenylephrine (PE, 1 µM) stimulated IP_3_R to induce sarcoplasmic reticulum Ca^2+^ release, resulting in transient vasoconstriction. The positive control 2-APB (100 µM, IP_3_R blocker) almost completely inhibited contraction (maximum contraction = 0.03 ± 0.02 g, *p* < 0.001), confirming the validity of the model. CA also significantly inhibited PE-induced contraction at concentrations of 5.29 µM and 84.62 µM (maximum contraction: 0.45 ± 0.06 g and 0.43 ± 0.03 g, respectively; *p* < 0.05). However, CA showed no significant effect at 21.15 µM (0.70 ± 0.05 g, *p* > 0.05), indicating an atypical concentration-independent response.

## 3. Discussion

### 3.1. Possible Pathway Involvement Based on Pharmacological Screening

This study provides a broad pharmacological screening of CA-induced vasorelaxation in isolated rat aortic rings. Combining the results shown in Figure 1, Figure 2, Figure 3 and Figure 4 and Table 1, the relaxation effect of CA appears to involve several pathways, including endothelium-dependent signalling, EDRF-related mechanisms, GPCR-related pathways, and potassium channel modulation. However, because the evidence is based mainly on pharmacological blockers, these results should be interpreted as pathway involvement rather than direct proof of receptor activation or direct potassium channel opening. Furthermore, this study did not directly detect downstream endothelial or vascular smooth muscle signaling mediators, including NO, cGMP, cAMP, prostacyclin, or other prostaglandin products. Therefore, the inhibitory effects observed after pretreatment with L-NAME, ODQ, methylene blue, indomethacin, atropine, propranolol, and potassium channel blockers should be interpreted as indirect pharmacological evidence supporting the involvement of these pathways, rather than definitive proof that CA directly activates endothelial receptors, GPCRs, or potassium channels.

### 3.2. Dependence on Endothelial Signaling

Compared with endothelium-intact aortic rings, CA-induced vasorelaxation was significantly weakened in endothelium-denuded rings (Figure 1, Table 1). Statistically significant differences between the two groups were observed within the cumulative concentration range of 79.33–163.95 µM. The inhibitor experiments shown in Figure 2 and Table 1 further support the conclusion that the vasodilatory effect of CA is largely dependent on endothelium-related factors.

### 3.3. Interactions with GPCRs

(Figure 3, Table 1) In isolated rat aortic rings, M3 receptors are expressed on endothelial cells and are coupled to Gq proteins. Activation of M3 receptors can increase endothelial intracellular Ca^2+^ concentration and promote NO formation through endothelial nitric oxide synthase. Therefore, inhibition by atropine may indicate involvement of muscarinic receptor-related endothelial signalling, but it does not prove that CA directly activates M3 receptors.

In the present study, pretreatment with atropine, a non-selective muscarinic receptor antagonist, attenuated CA-induced relaxation. These results suggest that muscarinic receptor-related endothelial signalling may partly contribute to CA-induced vasorelaxation.

In addition, propranolol is a non-selective β-adrenergic receptor antagonist. In the rat aorta, the β2-adrenergic receptor subtype is mainly expressed in vascular smooth muscle, is coupled to Gs proteins, and can increase intracellular cAMP levels to mediate vasorelaxation when activated [17,18].

In the present study, pretreatment with propranolol (1 µM) significantly inhibited CA-induced relaxation. These results suggest that beta-adrenergic receptor-related signalling may also contribute to the vasorelaxant effect of CA. However, further studies measuring cAMP levels or using subtype-selective antagonists are needed to confirm this pathway.

### 3.4. Co-Regulation of the K^+^ Channel Network

Based on the blocker data in Figure 4 and Table 1, KATP channels appeared to contribute strongly to CA-induced relaxation: glibenclamide, a selective KATP blocker, produced a marked inhibitory effect, reducing RMAX from 90.08% to 24.05%. This suggests that ATP-sensitive potassium channels may be involved in the relaxation response.

Calcium-dependent potassium channels can be classified into large-, intermediate-, and small-conductance types. Large-conductance calcium-activated potassium channels are voltage-dependent, whereas intermediate- and small-conductance channels are not; their blockers also differ [19]. TEA, a KCa blocker, also showed a marked inhibitory effect (RMAX decreased to 32.91%), suggesting the possible involvement of Ca^2+^-activated K^+^ channels.

Kir and Kv channels: BaCl2 (a non-selective Kir blocker) and 4-AP (a non-selective Kv blocker) also reduced the relaxation response. However, the overall concentration-response curve trend was closer to the PE control curve than to the EDRF-blocked groups, suggesting that Kir and Kv channels may contribute to CA-induced relaxation but are unlikely to be the dominant pathway.

This study provides a comparative assessment of the apparent contribution of four K^+^ channel types to CA-induced relaxation. Based on blocker sensitivity, the apparent order of contribution was KATP > KCa > Kir > Kv. This order should be interpreted cautiously because pharmacological blockers indicate pathway involvement but do not directly demonstrate channel opening.

### 3.5. Possible Involvement of IP3R-Mediated Calcium Release Rather than Direct VOCC Blockade

The comparison between KCl- and PE-precontracted aortic rings is informative. In KCl-precontracted rings (Figure 1, Table 1), the resting membrane potential is mainly determined by the transmembrane potassium ion concentration gradient. Elevated extracellular potassium reduces this gradient and decreases the absolute value of the resting membrane potential, causing partial depolarization. This depolarization activates voltage-operated calcium channels (VOCCs), promotes Ca^2+^ influx, increases intracellular Ca^2+^ concentration, and ultimately triggers vascular smooth muscle contraction [20].

In contrast, in PE-precontracted aortic rings, PE binds to α_1_-adrenergic receptors on vascular smooth muscle cells and activates the Gq protein-phospholipase C (PLC) signaling cascade. PLC activation generates IP_3_ and diacylglycerol (DAG). IP_3_ stimulates Ca^2+^ release from the sarcoplasmic reticulum, whereas DAG activates protein kinase C (PKC). The resulting increase in intracellular Ca^2+^ activates myosin light-chain kinase (MLCK), ultimately causing smooth muscle contraction [21].

Comparing the results obtained with KCl- and PE-precontracted rings suggests that CA-induced vasorelaxation may depend more on receptor-coupled signaling pathways than on direct VOCC blockade.

The calcium channel results (Figure 5a,b, Table 2) are consistent with the weak relaxation observed in KCl-precontracted rings and suggest that CA does not primarily act by directly blocking voltage-operated calcium channels. By contrast, partial inhibition of PE-induced contraction under calcium-free conditions suggests possible involvement of IP3R-mediated sarcoplasmic reticulum calcium release.

### 3.6. Atypical Concentration-Response Finding

As shown in Figure 5c and Table 2, under calcium-free conditions, PE induces IP3R-mediated sarcoplasmic reticulum Ca^2+^ release by activating α1-adrenergic receptors. CA significantly reduced PE-induced contractile responses at 5.29 µM and 84.62 µM, but no significant inhibition was observed at 21.15 µM, suggesting that its effect may not be a simple linear concentration-dependent relationship. This non-monotonic response may be related to multiple factors. First, biological variability may exist among different rats, such as differences in baseline vascular tone, PE response sensitivity, endothelial functional status, and smooth muscle contractility, thus affecting the extent of CA-mediated inhibition of PE-induced contraction. Second, tissue heterogeneity between aortic rings may also be involved; even from the same thoracic aorta, different segments may differ in endothelial integrity, receptor expression levels, smooth muscle layer status, and drug sensitivity. Third, this result may reflect a degree of biphasic pharmacology, meaning that low concentrations of CA may primarily affect receptor-coupled Ca^2+^ release, while higher concentrations of CA may further affect membrane-related signals, Ca^2+^ processing, or other regulatory mechanisms. Therefore, intermediate concentrations may not necessarily show a linearly enhanced inhibitory effect. Furthermore, receptor desensitization may also affect the results, as continuous or repeated PE stimulation may alter the sensitivity of α1-adrenergic receptor-related signals. Experimental variability, such as subtle differences in vascular ring preparation, basal tension stability, washing procedures, dosing intervals, and recording time points, may also lead to fluctuations in response between different concentration groups. In summary, the lack of significant inhibition in the 21.15 µM group is likely due to a combination of factors including individual animal differences, tissue heterogeneity, biphasic pharmacological effects, changes in receptor sensitivity, and experimental variability.

### 3.7. Integration of Anti-Inflammatory and Vasodilatory Effects

Indomethacin treatment significantly reduced the relaxant effect of CA. As a non-selective COX inhibitor, indomethacin may affect vascular responses by inhibiting COX-mediated PGI_2_ production. PGI_2_ binds to IP receptors on vascular smooth muscle cells and activates adenylyl cyclase (AC) through Gαs proteins, promoting the conversion of ATP to cAMP. Elevated cAMP activates PKA and triggers vasorelaxation. Therefore, reduced PGI_2_ synthesis after COX inhibition may impair the cAMP/PKA-dependent relaxation mechanism, thereby diminishing the vasodilatory effect [19].

The significant inhibition of CA-induced relaxation by indomethacin suggests that COX-derived prostanoid pathways may partly contribute to the vasorelaxant effect of CA. However, direct measurement of prostacyclin or other prostanoid mediators is needed to confirm this mechanism.

Although CA has been widely reported to have anti-inflammatory properties through inhibition of NF-kappaB [22,23,24], the present indomethacin data suggest that prostanoid-related signalling may also participate in its vascular response. This should be interpreted cautiously because prostaglandins such as PGI2 were not directly measured in the present study.

Previous studies have reported that CA may regulate oxidative stress, inflammation, and metabolic pathways, including Nrf2, AMPK, and NF-kappaB-related signalling. These reported properties may be relevant to vascular dysfunction, but direct confirmation in the present aortic ring model will require further molecular assays.

### 3.8. Dose–Response Relationship and Translational Considerations

The results of this study indicated that the vasodilatory effect of CA was concentration-dependent, with the maximum effect observed at the highest cumulative concentration (163.95 µM; RMAX = 90.08 ± 7.33%). However, this concentration should not be directly interpreted as a clinically achievable plasma concentration, as the isolated aortic ring model involves direct tissue exposure and does not reflect in vivo absorption, distribution, metabolism, or elimination. Furthermore, previous studies have reported that high concentrations of CA or its derivatives may have cytotoxic effects [25,26]. Therefore, the results of this study should be considered preliminary ex vivo pharmacological evidence, and further pharmacokinetic, toxicological, and in vivo hemodynamic studies are needed to determine the therapeutic window of CA before considering clinical translation. Reducing toxicity while maintaining efficacy is therefore a key issue in the development of CA.

In recent years, nanodrug delivery systems have been explored as a possible strategy to improve the delivery and bioavailability of poorly soluble natural compounds. For CA, future formulation studies may help to improve solubility, reduce off-target exposure, and support controlled release. However, this remains a future research direction and was not examined in the present study.

### 3.9. Research Prospects and Future Validation

Overall, CA, as a natural triterpene compound, warrants further investigation for its vascular pharmacological properties. However, several studies are still needed before any clinical significance can be considered. Future research should include direct detection of NO production, cGMP and cAMP levels, and prostaglandin mediators such as 6-keto-PGF1α; it should also incorporate receptor binding assays, subtype-selective antagonist studies, and patch-clamp electrophysiological experiments to confirm whether CA directly regulates GPCR-related pathways or potassium channels. Furthermore, in vivo hemodynamic evaluations, pharmacokinetic assessments, safety testing, and validation in hypertension models are required.

## 4. Limitations

This study has several limitations. First, the findings were obtained from isolated rat aortic rings and therefore should be considered preliminary ex vivo evidence. Second, this study’s explanation of the mechanism of action is primarily based on pharmacological blockers. While these blockers help identify potentially involved pathways, they do not pinpoint the direct molecular targets of CA. This study did not directly detect NO, cGMP, cAMP, prostacyclin or its stable metabolite 6-keto-PGF1α, receptor binding activity, or potassium channel currents. Third, CA showed a non-monotonic response in the IP3R-mediated calcium-release experiment, and this observation requires further verification. Finally, in vivo antihypertensive activity, pharmacokinetics, and safety were not assessed in the present study.

## 5. Materials and Methods

### 5.1. Chemical Materials (Table 3)

The reagents used in this study included various antagonists, components of Krebs–Henseleit physiological solution, and the test compound (Table 3).

**Table 3:** Sources of chemical products.

Product	Supplier	Country
Corosolic acid	MedChemExpress	USA
Propranolol, atropine, 2-APB, indomethacin, glibenclamide, L-NAME, ODQ, BaCl_2_, CaCl_2_, TEA, 4-AP, DMSO, and Tween 80	Sigma Aldrich	USA
PE, ACh, and nifedipine	Acros Organics	Geel, Belgium
MB	Pro-medipharm Sdn. Bhd.	
potassium chloride	R&M Chemicals	Malaysia
EGTA	Calbiochem	Germany

### 5.2. Animals

Male Sprague-Dawley rats, aged eight to ten weeks and weighing between 200 and 250 g, were utilized in this study. These animals were specific pathogen-free (SPF) grade. Housing conditions included a constant ambient temperature of 25 °C and a relative humidity range of 40% to 70%. The rats received a standard laboratory diet and were kept on a 12-h light/dark cycle schedule. All experimental protocols were conducted in strict adherence to the animal care and usage guidelines established by Universiti Sains Malaysia.

### 5.3. Preparation of Antagonists

Various antagonists were prepared in specific solvents. Phenylephrine (PE, 1 µM), acetylcholine (ACh, 1 µM), atropine (1 µM), barium chloride (BaCl_2_, 10 µM), L-NAME (10 µM), methylene blue (MB, 10 µM), propranolol (1 µM), tetraethylammonium (TEA, 1 mM), and 4-aminopyridine (4-AP, 1 mM) were individually dissolved in 2 milliliters of distilled water. Conversely, glibenclamide (10 µM), indomethacin (10 µM), nifedipine (1 µM), ODQ (1 µM), and 2-APB (100 µM) were prepared using a mixture of 1% Tween 80 and distilled water.

### 5.4. Preparation of Aortic Vascular Rings

Rats were euthanized by carbon dioxide (CO_2_) inhalation. The thoracic cavity was rapidly opened to expose the ascending aorta. The thoracic aorta was then carefully isolated intact from the aortic root (starting at the left ventricle) to the diaphragm.

A Petri dish was prepared with 10 mL of Krebs–Henseleit physiological solution (KPS; 118.0 mM NaCl, 4.7 mM KCl, 25.0 mM NaHCO_3_, 1.8 mM CaCl_2_, 1.2 mM KH_2_PO_4_, 1.2 mM MgSO_4_, and 11.0 mM glucose; pH 7.4). The solution was continuously aerated with 95% O_2_ and 5% CO_2_. The isolated aortic segment was immediately transferred to this dish.

Connective tissue adhering to the aorta was carefully removed in the Petri dish until the vascular outline was clearly visible. The cleaned aorta was cut into eight rings, each approximately 2–3 mm long.

A segment of aortic ring was passed successively over the hooks fixed to the L-shaped stent and the L-shaped support frame. During installation, the hooks were positioned above the L-shaped stent, and both were passed coaxially through the lumen of the aortic ring without touching each other. The assembled device was placed in the organ bath, and the distal end of the L-shaped stent was firmly fixed. The free end of the suture connected to the hooks was passed through the central hole of the metal plate below the force-displacement transducer (FT03; GRASS Instruments/Grass Technologies, West Warwick, RI, USA), tied tightly with a surgical knot on the lower surface of the transducer, and trimmed. The vascular tension signal was amplified by the Quad Bridge Amp, digitized by the PowerLab 26T data acquisition system, and recorded and displayed in real time using LabChart 7.3.8 software. Notes: The suture must not contact the L-shaped stent at any point. The hook and L-shaped stent must pass coaxially through the aortic ring (i.e., along the same central axis), with the hook above and the L-shaped stent below, and there must be no physical contact between the two [27,28,29].

### 5.5. Organ Bath Conditions and Replicate Handling

The organ bath is aerated with a mixture of 95% oxygen and 5% carbon dioxide for at least 15–20 min to saturate and stabilize the pH at 7.4. Each organ bath contains 10 mL of Kreb’s solution. After inserting the vascular ring, wash the ring three times for 10 min each time.

### 5.6. Corosolic Acid Preparation

Corosolic acid was dissolved in 0.0625% DMSO and diluted with distilled water to prepare a 2 mL stock solution (4 mg/mL). The stock solution was serially diluted to prepare working solutions, and 100 µL of each working solution was added cumulatively to the 10 mL organ bath. The final cumulative bath concentrations of CA were 5.29, 15.87, 37.02, 79.33, and 163.95 µM.

### 5.7. Aortic Ring Functional Testing and Screening

The liquid inlet valve of the organ bath was opened, and all eight baths were rinsed once with KPS solution. KPS solution was then added to each bath. The gas mixture (95% O_2_ and 5% CO_2_) was introduced, and gas outflow from each bath was visually checked. The gas valves were adjusted until uniform and consistent bubbles were produced at all outlets. Ventilation was continued for at least 15–20 min. The prepared aortic rings were then carefully placed into the baths and connected to the apparatus.

The aortic rings were rinsed with KPS solution three times, with each rinse lasting 10 min. Concentrated phenylephrine (PE) solution (100 µL) was added to a 10 mL organ bath to obtain a final concentration of 1 µM and maintained for 10 min. Concentrated acetylcholine (ACh) solution (100 µL) was then added to obtain a final concentration of 1 µM and maintained for 5 min. For endothelium-intact vascular rings, successful functional verification required: (i) a PE-induced contraction amplitude ≥ 1.6 g; and (ii) an ACh-induced relaxation amplitude of at least 60% of the PE-induced contraction amplitude. For endothelium-denuded experimental groups, the criterion for successful endothelial removal was an ACh-induced relaxation amplitude <10% of the PE-induced contraction amplitude or no relaxation response.

After functional assessment, the aortic rings were rinsed again with KPS solution three times, with each rinse lasting 10 minutes [27,28,29].

### 5.8. Preliminary Screening of the Vasodilatory Effect of CA on Endothelium-Intact Aortic Rings

After the equilibration steps, PE (final concentration 1 µM) was added to the bath to precontract the vascular rings. The rings were maintained for 30 min until a stable vascular tension plateau was obtained. CA was then added cumulatively at 20 min intervals (100 µL of each working solution; 0.25–4 mg/mL), resulting in final cumulative bath concentrations of 5.29–163.95 µM [28,30,31]. Changes in vascular tension were continuously recorded using the organ bath recording system, and concentration-response curves were constructed accordingly.

### 5.9. Vasodilatory Effect of CA on Endothelium-Denuded Aortic Rings

The endothelium of the vascular rings was mechanically removed using cotton swabs, and successful removal was verified by an ACh-induced relaxation response of less than 10% of the PE-induced contraction. The endothelium-denuded rings were then precontracted with PE (final concentration 1 µM) for 30 min until a stable tension plateau was reached. CA was added cumulatively every 20 min, increasing the final concentration from 5.29 µM to 163.95 µM [27,28,29]. The vasodilatory responses were then compared with those of endothelium-intact rings.

### 5.10. Vasodilatory Effect of Corosolic Acid on Isolated Aortic Rings Precontracted with Potassium Chloride

PE (1 µM) was replaced with 80 mM KCl to precontract endothelium-intact vascular rings for 30 min until a stable tension plateau was obtained. CA was then added at 20 min intervals using the same cumulative dosing method, increasing the final concentration from 5.29 µM to 163.95 µM [27,28,29]. The resulting concentration-response curve was compared with that obtained from PE-precontracted rings.

### 5.11. Relationship Between CA-Induced Relaxation of Aortic Rings and EDRFs

To investigate whether CA induces vasorelaxation through EDRF-related signaling pathways, specific pharmacological inhibitors were used to block different sites in this pathway.

In the independent experimental group, the following inhibitors were added to reach the specified final concentrations and incubated for 20 min to achieve pathway blockade: 10 μM L-NAME (nitric oxide synthase inhibitor), 1 μM ODQ (selective inhibitor of soluble guanylate cyclase), 10 μM methylene blue (inhibitor of NO–cGMP signaling), and 10 μM indomethacin (non-selective cyclooxygenase inhibitor). After inhibitor incubation, the vascular rings were precontracted with PE for 30 min. CA was then added cumulatively at predetermined concentrations at 20 min intervals [28,30,31]. The vasodilatory responses in each inhibitor-treated group were compared with those in the control group without inhibitors.

### 5.12. Relationship Between CA-Induced Relaxation of Aortic Rings and GPCRs

To investigate whether CA mediates vasorelaxation through GPCR-related signaling pathways, specific pharmacological inhibitors were used to selectively block different sites in this pathway.

In the independent experimental group, the following inhibitors were added to reach the specified final concentrations and incubated for 20 min to achieve effective blockade: 1 µM atropine (a non-selective muscarinic receptor antagonist) and 1 μM propranolol (a non-selective β-adrenergic receptor antagonist). After inhibitor incubation, the vascular rings were precontracted with PE for 30 min. CA was then added cumulatively at predetermined concentrations at 20 min intervals [28,29,31]. The vasodilatory responses in the inhibitor-treated groups were compared with those in the control group without inhibitor pretreatment.

### 5.13. Relationship Between CA-Induced Relaxation of Aortic Rings and Potassium Channels

To determine whether CA induces vasorelaxation through potassium channel-related signaling pathways, specific pharmacological inhibitors were used to selectively block different potassium channel subtypes.

In the independent experimental group, the following inhibitors were added (all at the specified final concentrations) and incubated for 20 min to achieve effective blockade: TEA (1 mM, a non-selective blocker for calcium-activated potassium channels (KCa)), 4-AP (1 mM, a non-selective blocker for voltage-gated potassium channels (Kv)), BaCl_2_ (10 µM, a non-selective blocker for inwardly rectifying potassium channels (Kir)), and glibenclamide (10 µM, a selective blocker for ATP-sensitive potassium channels (KATP)). After inhibitor incubation, the vascular rings were precontracted with PE for 30 min. CA was then added cumulatively at predetermined concentrations at 20 min intervals [28,29,31]. The vasodilatory responses in each inhibitor-treated group were compared with those in the control group without inhibitors.

### 5.14. Effect of CA Under Extracellular Calcium-Mediated Vascular Contraction Conditions

To investigate whether CA induces aortic vasorelaxation through the voltage-operated calcium channel (VOCC) pathway, experiments were divided into three groups: a negative control group, a positive control group, and a CA treatment group.

After the functional integrity of the isolated aortic ring had been confirmed, the ring was placed in KPS solution for 10 min to stabilize the baseline tension. The ring was then incubated in high-potassium, calcium-free KPS solution containing 0.2 mM EGTA for 15 min. It was subsequently washed twice with high-potassium, calcium-free KPS solution (91.04 mM NaCl, 50.0 mM KCl, 11.9 mM NaHCO_3_, 1.05 mM MgSO_4_, and 5.5 mM glucose; pH 7.4), with each wash lasting 10 min. The aortic ring endothelium remained intact throughout the experiment.

Negative control group: CaCl_2_ was added cumulatively to the bath chamber at 3 min intervals, with final concentrations ranging from 0.01 mM to 10 mM. Positive control group: Vascular rings were pre-incubated for 20 min with nifedipine, a selective L-type VOCC blocker, at final concentrations of 0.1 µM, 0.3 µM, or 1 µM, and CaCl_2_ was then added using the same protocol as in the negative control group. CA treatment group: Nifedipine was replaced with Corosolic acid, and vascular rings were incubated with CA at final concentrations of 5.29 µM, 21.15 µM, or 84.62 µM for 20 min before the same CaCl_2_ concentration-response experiment was performed. The aortic ring endothelium was kept intact in this group [28,29,30].

The cumulative concentration-response curves obtained from each group were quantitatively compared, and the negative control data were used as the reference for normalization and statistical analysis.

### 5.15. Relationship Between CA-Induced Vasorelaxation of Aortic Rings and Sarcoplasmic Reticulum Calcium Release

To investigate whether CA promotes aortic vasorelaxation by inhibiting sarcoplasmic reticulum calcium release, experiments were divided into a negative control group, a positive control group, and a CA treatment group. After the integrity of the vascular rings had been confirmed, the tissues were placed in KPS solution for 10 min to stabilize baseline tension. The rings were then incubated in high-potassium, calcium-free KPS solution containing 0.2 mM EGTA for 15 min. Subsequently, the rings were washed twice with high-potassium, calcium-free KPS solution without EGTA (same composition as above), with each wash lasting 10 min.

Negative control group: PE was added to the organ bath to achieve a final concentration of 1 µM and maintained for 20 min. Positive control group: Rings were first incubated with 2-aminoethoxydiphenyl borate (2-APB, 100 µM) for 20 min, and PE (1 µM) was then added for another 20 min. CA treatment group: 2-APB was replaced with CA, and rings were pre-incubated with CA at final concentrations of 5.29 µM (low), 21.15 µM (medium), or 84.62 µM (high) for 20 min before PE (1 µM) was added for another 20 min. The aortic ring endothelium was removed in this group [28,29,30].

The response curves obtained from each group were compared and analyzed using the negative control group as the reference standard.

### 5.16. Statistical Analysis

All experimental data were recorded and analysed using appropriate statistical software. Results are expressed as mean ± S.E.M. Unless otherwise stated, *n* should represent the number of animals, with one aortic ring per animal used for each treatment condition. If multiple rings from the same animal were used in the same treatment condition, values from that animal should be averaged before statistical analysis to avoid treating technical replicates as independent biological replicates.

The statistical analysis was calculated using the one-way analysis of variance (one-way ANOVA) using the SPSS Version 18 software. All the tests were two-tailed, and the significance values were set as *p* < 0.05.

The maximum relaxation (RMAX) and potency (pD_2_) of CA were calculated using the following equations. pD_2_ was calculated only when the concentration-response curve reached at least 50% relaxation. For incomplete curves with RMAX < 50%, pD_2_ was reported as not determined (ND).

RMAX = (b − a)/b × 100%

pD_2_ = −log EC_50_

where b = contraction force (g) after 30 min of PE incubation; a = contraction force (g) after 20 min of incubation with the final dose of CA; and EC_50_ = half-maximal effective concentration. Concentration-response curves should be fitted using nonlinear regression where possible.

## 6. Conclusions

In this study, CA induced concentration-dependent relaxation in PE-precontracted endothelium-intact rat aortic rings. The response was markedly reduced by endothelial removal, EDRF pathway inhibition, GPCR-related antagonists, and potassium channel blockers. CA did not significantly inhibit VOCC-mediated Ca^2+^ influx but partially reduced PE-induced contraction under calcium-free conditions, suggesting possible involvement of IP3R-mediated calcium release. Overall, these findings provide preliminary ex vivo evidence that CA may have vasorelaxant properties through multiple pathway-related mechanisms. Further molecular, electrophysiological, and in vivo studies are required before any therapeutic relevance can be established.

## Figures and Tables

**Table 1:** Comparison of maximum relaxation and pD_2_ of CA-induced vasorelaxation under different conditions.

Treatment	*N*	Concentration	RMAX (%)	pD_2_
Screening				
Endothelium-intact	6	-	90.08 ± 7.33	4.21 ± 0.08
Endothelium-denuded	6	-	17.35 ± 8.61 ***	ND
KCl-precontracted	6	80 mM	23.15 ± 6.17 ***	ND
EDRFs and NO signaling pathway				
L-NAME	6	10 µM	23.38 ± 3.15 ***	ND
ODQ	6	1 µM	11.97 ± 6.82 ***	ND
Methylene blue	6	10 µM	16.46 ± 2.33 ***	ND
Indomethacin	6	10 µM	35.19 ± 10.51 ***	ND
GPCRs				
Propranolol	6	1 µM	30.23 ± 9.25 ***	ND
Atropine	6	1 µM	44.43 ± 5.22 ***	ND
Potassium channels				
TEA	6	1 mM	32.91 ± 2.44 ***	ND
4-AP	6	1 mM	52.53 ± 5.53 ***	3.88 ± 0.10
BaCl_2_	6	10 µM	38.21 ± 5.40 ***	ND
Glibenclamide	6	10 µM	24.05 ± 6.19 ***	ND

Notes: Results are expressed as mean ± S.E.M.; significance is indicated as * *p* < 0.05, ** *p* < 0.01, and *** *p* < 0.001 compared with the endothelium-intact PE-precontracted control unless otherwise indicated. All concentrations are final concentrations in the organ bath. *n* = number of aortic rings/animals as defined in the Methods; RMAX = maximum relaxation; pD_2_ = potency index (−log EC_50_); ND = not determined because the relaxation curve did not reach 50%; EDRFs = endothelium-derived relaxing factors; NO = nitric oxide; GPCRs = G protein-coupled receptors.

**Table 2:** Comparison of maximum contraction values among treatments in calcium channel mechanism studies.

Treatment	*N*	Concentration	Maximin Contraction (g)
VOCC			
Negative control	6	-	0.33 ± 0.09
Nifedipine	6	0.1 µM	0.10 ± 0.02
Nifedipine	6	0.3 µM	0.07 ± 0.02
Nifedipine	6	1 µM	0.01 ± 0.02 *
Corosolic acid	6	5.29 µM	0.22 ± 0.14
Corosolic acid	6	21.15 µM	0.31 ± 0.10
Corosolic acid	6	84.62 µM	0.24 ± 0.06
IP3R			
Negative control	6	-	0.61 ± 0.03
2-APB	6	100 µM	0.03 ± 0.02 ***
Corosolic acid	6	5.29 µM	0.45 ± 0.06 *
Corosolic acid	6	21.15 µM	0.70 ± 0.05
Corosolic acid	6	84.62 µM	0.43 ± 0.03 *

Notes: Results are expressed as mean ± S.E.M.; significance is indicated as * *p* < 0.05, ** *p* < 0.01, and *** *p* < 0.001 compared with the negative control group. All concentrations are final concentrations in the organ bath. *n* = number of aortic rings; VOCC = voltage-operated calcium channel; IP_3_R = inositol 1,4,5-trisphosphate receptor.

## Data Availability

Data available in a publicly accessible repository.

## References

[1] Li L., Su X.-L., Bai T.-T., Qin W., Li A.-H., Liu Y.-X., Wang M., Wang J.-K., Xing L., Li H.-J. (2022). New paeonol derivative C302 reduces hypertension in spontaneously hypertensive rats through endothelium-dependent and endothelium-independent vasodilation. Eur. J. Pharmacol..

[2] Ebinger J.E., Kauko A., Vaura F., Hage P., Sundstrom J., Joung S.Y., Cheng S., Niiranen T.J., FinnGen (2026). GWAS and Replication Analysis of Apparent Treatment-Resistant Hypertension. Hypertension.

[3] Lu L.-Q., Li A.N.-S., Li A.M.-R., Peng J.-Y., Tang L.-J., Luo X.-J., Peng J. (2023). DL-3-n-butylphthalide improves the endothelium-dependent vasodilation in high-fat diet-fed ApoE^−/−^ mice via suppressing inflammation, endothelial necroptosis and apoptosis. Eur. J. Pharmacol..

[4] Maruhashi T., Soga J., Fujimura N., Idei N., Mikami S., Iwamoto Y., Kajikawa M., Matsumoto T., Hidaka T., Kihara Y. (2013). Nitroglycerine-Induced Vasodilation for Assessment of Vascular Function A Comparison with Flow-Mediated Vasodilation. Arterioscler. Thromb. Vasc. Biol..

[5] Demirel S. (2022). Rosa damascena Miller essential oil relaxes rat thoracic aorta through the NO-cGMP-dependent pathway. Prostaglandins Other Lipid Mediat..

[6] Gonzalez M., Clayton S., Wauson E., Christian D., Tran Q.-K. (2025). Promotion of nitric oxide production: Mechanisms, strategies, and possibilities. Front. Physiol..

[7] Hu Y., Chen M., Wang M., Li X. (2022). Flow-mediated vasodilation through mechanosensitive G protein-coupled receptors in endothelial cells. Trends Cardiovasc. Med..

[8] Murphy T.V., Sandow S.L. (2019). Agonist-evoked endothelial Ca^2+^ signalling microdomains. Curr. Opin. Pharmacol..

[9] Da Silva-Junior E.F., Silva L.R. (2022). Multi-target Approaches of Epigallocatechin-3-O-gallate (EGCG) and its Derivatives against Influenza Viruses. Curr. Top. Med. Chem..

[10] Talati Y.K., Gaikwad A.B. (2024). A therapeutic approach to identify leading molecules from natural products and therapeutic targets in CKD by network pharmacology. Pharmanutrition.

[11] Gaur P.K., Chauhan V., Chaturvedi S., Hashmi R., Sharma P., Mishra R. (2025). Development and Evaluation of Corosolic Acid Nanoemulsion for Potentiation Activity Against MCF-7 Breast Cancer Cells. J. Surfactants Deterg..

[12] Uddin M.B., Shipon M.N.H., Suma R.B., Rahman M.A., Alkhathami A.G., Mia E., Khalipha A.B., Akter K., Al Hasan M.S. (2026). Anticancer Activity of Corosolic Acid with Botanical Sources, Biopharmaceutical Profile, Mechanistic Insight, Toxicity, and Clinical Evidence. Phytochem. Anal..

[13] Singh T.R., Ezhilarasan D. (2021). *Lagerstroemia speciosa* (L.) Pers., ethanolic extract attenuates simultaneously administered isoniazid- and dapsone-induced hepatotoxicity in rats. J. Food Biochem..

[14] Xu S., Wang G., Peng W., Xu Y., Zhang Y., Ge Y., Jing Y., Gong Z. (2019). Corosolic acid isolated from Eriobotrya japonica leaves reduces glucose level in human hepatocellular carcinoma cells, zebrafish and rats. Sci. Rep..

[15] Sharma H., Kumar P., Deshmukh R.R., Bishayee A., Kumar S. (2018). Pentacyclic triterpenes: New tools to fight metabolic syndrome. Phytomedicine.

[16] Wang Z.-P., Che Y., Zhou H., Meng Y.-Y., Wu H.-M., Jin Y.-G., Wu Q.-Q., Wang S.-S., Yuan Y. (2020). Corosolic acid attenuates cardiac fibrosis following myocardial infarction in mice. Int. J. Mol. Med..

[17] Koch T., Gloddek B., Gutzke S. (1992). Binding-sites of atrial-natriuretic-peptide (anp) in the mammalian cochlea and stimulation of cyclic-gmp synthesis. Hear. Res..

[18] Murasaki O., Kaibara M., Nagase Y., Mitarai S., Doi Y., Sumikawa K., Taniyama K. (2003). Site of action of the general anesthetic propofol in muscarinic M1 receptor-mediated signal transduction. J. Pharmacol. Exp. Ther..

[19] Loh Y.C., Tan C.S., Ch’ng Y.S., Yeap Z.Q., Ng C.H., Yam M.F. (2018). Overview of the Microenvironment of Vasculature in Vascular Tone Regulation. Int. J. Mol. Sci..

[20] Hattori T., Wang P.-L. (2006). Involvement of Na^+^-K^+^-2Cl^−^ cotransporters in hypertonicity-induced rise in intracellular calcium concentration. Int. J. Neurosci..

[21] Fatatis A., Holtzclaw L.A., Avidor R., Brenneman D.E., Russell J.T. (1994). Vasoactive-intestinal-peptide increases intracellular calcium in astroglia—Synergism with alpha-adrenergic receptors. Proc. Natl. Acad. Sci. USA.

[22] Cheng Q.-L., Li H.-L., Li Y.-C., Liu Z.-W., Guo X.-H., Cheng Y.-J. (2017). CRA (Corosolic Acid) isolated from *Actinidia valvata* Dunn. Radix induces apoptosis of human gastric cancer cell line BGC823 in vitro via down-regulation of the NF-κB pathway. Food Chem. Toxicol..

[23] Zhang J.-X., Feng W.-J., Liu G.-C., Ma Q.-Q., Li H.-L., Gao X.-Y., Liu H.-Z., Piao G.-C., Yuan H.-D. (2020). Corosolic Acid Attenuates Hepatic Lipid Accumulation and Inflammatory Response via AMPK/SREBPs and NF-κB/MAPK Signaling Pathways. Am. J. Chin. Med..

[24] Liu G., Cui Z., Gao X., Liu H., Wang L., Gong J., Wang A., Zhang J., Ma Q., Huang Y. (2021). Corosolic acid ameliorates non-alcoholic steatohepatitis induced by high-fat diet and carbon tetrachloride by regulating TGF-β1/Smad2, NF-κB, and AMPK signaling pathways. Phytother. Res..

[25] Petchimuthu P., Kunjiappan S., Pandian S.R.K., Sankaranarayanan M., Sundar K. (2025). Sorghum Grain-Derived Kafirin Nanoparticles For Effective Delivery of Corosolic Acid into Breast Cancer Cells for Potential Treatment of Breast Cancer. J. Clust. Sci..

[26] Heise N.V., Hoenke S., Serbian I., Csuk R. (2022). An improved partial synthesis of corosolic acid and its conversion to highly cytotoxic mitocans. Eur. J. Med. Chem. Rep..

[27] Tew W.Y., Tan C.S., Asmawi M.Z., Yam M.F. (2020). Underlying mechanism of vasorelaxant effect exerted by 3,5,7,2′,4′- pentahydroxyflavone in rats aortic ring. Eur. J. Pharmacol..

[28] Tew W.Y., Tan C.S., Yan C.S., Loh H.W., Wang X., Wen X., Wei X., Yam M.F. (2024). Mechanistic study on vasodilatory and antihypertensive effects of hesperetin: Ex vivo and in vivo approaches. Hypertens. Res..

[29] Tew W.Y., Tan C.S., Yan C.S., Loh H.W., Wen X., Wei X., Yam M.F. (2023). Evaluation of vasodilatory effect and antihypertensive effect of chrysin through in vitro and sub-chronic in vivo study. Biomed. Pharmacother..

[30] Wang X., Xu X., Tew W.Y., Ouyang L., Yang X., Loh H.W., Xu W., Xu W., Yam M.F. (2025). In Vivo Antihypertensive and Ex Vivo Vasodilatory Studies of Taxifolin. Pharmaceuticals.

[31] Yam M.F., Tew W.Y., Tan C.S., Loh H.W., Qiu Q., Yan C.S., Xu X., Ouyang L., Zhou R., Yap Y.P. (2025). Mechanistic insights into the synergistic vasodilatory actions of eupatorin, sinensetin, and 3′-hydroxy-5,6,7,4′-tetramethoxyflavone in ex vivo aortic ring model and their antihypertensive efficacy in in vivo rat model. Hypertens. Res..

